# Imaging of Carotid Stenosis: Where Are We Standing? Comparison of Multiparametric Ultrasound, CT Angiography, and MRI Angiography, with Recent Developments

**DOI:** 10.3390/diagnostics14161708

**Published:** 2024-08-06

**Authors:** Emanuele David, Hektor Grazhdani, Lorenzo Aliotta, Livio Maria Gavazzi, Pietro Valerio Foti, Stefano Palmucci, Corrado Inì, Francesco Tiralongo, Davide Castiglione, Maurizio Renda, Patrizia Pacini, Chiara Di Bella, Carmen Solito, Silvia Gigli, Alessandro Fazio, Rita Bella, Antonio Basile, Vito Cantisani

**Affiliations:** 1 Department of Medical Surgical Sciences and Advanced Technologies “GF Ingrassia”, University Hospital Policlinic “G. Rodolico-San Marco”, 95125 Catania, Italy; lore.aliotta@gmail.com (L.A.); gavazziliviomaria@gmail.com (L.M.G.); pietrofoti@hotmail.com (P.V.F.); spalmucci@unict.it (S.P.); corrado.ini@gmail.com (C.I.); tiralongofrancesco91@hotmail.it (F.T.); davidegiuseppecastiglione@gmail.com (D.C.); fazio.alessandro1993@gmail.com (A.F.); rbella@unict.it (R.B.); basile.antonello73@gmail.com (A.B.); 2 Department of Translational and Precision Medicine, “Sapienza” University of Rome, 00185 Rome, Italy; 3 Klinika Dani, ENI nr 13, 1010 Tiranë, Albania; he1graz@gmail.com; 4 Department of Radiological Sciences, Oncology and Pathology, Policlinico Umberto I, Sapienza University of Rome, 00161 Rome, Italy; maurizio.renda@uniroma1.it (M.R.); patry.shepsut91@gmail.com (P.P.); chiaradibella30@gmail.com (C.D.B.); solito.1014926@studenti.uniroma1.it (C.S.); vito.cantisani@uniroma1.it (V.C.); 5 Department of Diagnostic Imaging, Sandro Pertini Hospital, Via dei Monti Tiburtini, 385, 00157 Rome, Italy; adrenalina_1@hotmail.it

**Keywords:** multiparametric ultrasound, magnetic resonance imaging, computed tomography angiography, carotid stenosis, new technologies

## Abstract

Atherosclerotic disease of the carotid arteries is a crucial risk factor in predicting the likelihood of future stroke events. In addition, emerging studies suggest that carotid stenosis may also be an indicator of plaque load on coronary arteries and thus have a correlation with the risk of acute cardiovascular events. Furthermore, although in symptomatic patients the degree of stenosis is the main morphological parameter studied, recent evidence suggests, especially in asymptomatic patients, that plaque vulnerability should also be evaluated as an emerging and significant imaging parameter. The reference diagnostic methods for the evaluation of carotid stenosis are currently ultrasonography, magnetic resonance imaging (MRI), and computed tomography angiography (CTA). In addition, other more invasive methods such as 123I-metaiodobenzylguanidine (MIBG) scintigraphy and PET-CT, as well as digital subtraction angiography, can be used. Each method has advantages and disadvantages, and there is often some confusion in their use. For example, the usefulness of MRI is often underestimated. In addition, implementations for each method have been developed over the years and are already enabling a significant increase in diagnostic accuracy. The purpose of our study is to make an in-depth analysis of all the methods in use and in particular their role in the diagnostic procedure of carotid stenosis, also discussing new technologies.

## 1. Introduction

Nowadays, about 10–20% of ischemic stroke patients have an underlying atherosclerotic carotid disease. Several studies have revealed that about 25% of patients with atheromatic carotid stenosis reported a transient ischemic attack (TIA) prior to the disabling ischemic event. Therefore, atherosclerotic carotid disease represents a determining risk factor in predicting the future stroke event probability [1].

In addition, given the systemic nature of atherosclerosis, emerging studies suggest that carotid stenosis may also be an indicator of plaque load on coronary arteries and thus have a correlation with the risk of acute cardiovascular events, although this is not the subject of this review [2].

Atherosclerosis is a chronic vasal pathology characterized by the formation of fat streaks in the arterial walls that develop progressively in a so-called atheroma, a vascular plaque growing and occluding the arterial lumen. The clinical consequences of plaques vary depending on their location, stenosis degree, and growth rate [3].

The reference diagnostic methods for the evaluation of carotid stenosis are currently ultrasonography, magnetic resonance imaging (MRI), and computed tomography angiography (CTA) [4].

In addition, 123I-metaiodobenzylguanidine (MIBG) scintigraphy and PET-TC as well as digital subtraction angiography can be also performed [5].

The diagnostic sensitivity for carotid stenosis of the above imaging techniques fluctuates between 31% and 85%, while the specificity varies between 54% and 87% (for CE-MRA, for stenosis of 50–69%); these reported values are only reliable for luminal stenosis greater than 50% [6].

Therefore, a radiological evaluation of carotid disease is fundamental for a suitable stratification of stroke risk in affected patients. Although, in symptomatic patients, the stenosis degree is the main morphological parameter studied, recent evidence suggests, especially in asymptomatic patients, assessing even plaque vulnerability as an emerging significant imaging parameter [7].

Morphology and composition, in fact, influence plaque vulnerability; the radiologist’s attention focuses on finding new plaque features, such as intraplaque bleeding (IPH), ulceration, neovascularization, fibrous cap thickness, and the presence of a lipid necrotic core (LRNC). These entities appear to be responsible for the increased risk of plaque rupture and atherothrombotic and athero-embolic phenomena and are used, together with the degree of stenosis, for the stratification of patients for elective endovascular treatment [8].

## 2. Multiparametric Ultrasound

Carotid ultrasound (US) represents the first imaging modality for the study of carotid atheroma disease, offering the possibility of investigating wall morbidity to predict the risk of plaque complications at an early stage of the disease [9].

We have several ultrasound imaging techniques such as duplex ultrasound, contrast-enhanced ultrasound (CEUS), 3D ultrasound, and innovative ultra-fast vector flow ultrasound [9,10,11,12].

These techniques have the advantage of being rapid, noninvasive, time-resolved, inexpensive, and radiation-free instrumental investigations, but they are operator-dependent and do not allow for the assessment of the internal carotid artery (ICA) intracranial segments.

### 2.1. Standard Carotid US

B-mode US represents a first-level survey and allows for a morphological plaque changes evaluation through an IMT (thickness of the medium–intimal complex) study, considering the relative degree of luminal stenosis and ulcerative plaque phenomena that represent strong predictors for future embolic events risk. In particular, echogenic plaque variations allow for distinguishing calcific and hyperechoic components from soft and hypoechoic components, as predominantly soft plaques are highly susceptible to shear stress and therefore are a greater risk of future degenerative changes and represent a serious independent risk factor for stroke [9].

This type of evaluation, which uses high-frequency linear probes, allows for the assessment of plaque stenosis by the following means:

- North American Symptomatic Carotid Endarterectomy Trial (NASCET): comparison of the stenotic segment with the normal distal diameter of the post-stenotic ICA [13];
- European Carotid Surgery Trial (ECST): comparison of the diameter of the stenotic area with the normal diameter of the carotid bulb [14];
- Common Carotid (CC): measurement of the residual lumen diameter in the most stenotic portion of the artery and subsequent comparison with the lumen diameter of the proximal common carotid artery (CCA) [15].

The NASCET method underestimates the degree of stenosis compared with the ECST one.

Color Doppler US and the spectral Doppler mode add the possibility of a functional plaque study by investigating blood flow parameters such as flow direction and rate, which are encoded and represented on the monitor using color maps. In particular, the “peak of systolic speed (PVS)”, calculated through the spectral mode, is the main parameter used for the quantification of the carotid stenosis degree and is very reliable in cases of severe stenosis [6].

In the case of a healthy vase study, a color Doppler box must be placed in the center of the vase, parallel to the vase walls and distant from any coiling and kinking; otherwise, the color box should be placed in the most stenotic luminal section parallel to the blood flow.

Generally, color box sizes between 2 and 3 mm are used, but in the presence of severe carotid stenosis or strongly hyperechoic plaques, it may be necessary to enlarge it for detecting even minimal flows. The color gain should be adjusted to reach only the intimal coating, without affecting the plaque rating [16]. We emphasize, however, that very recent studies suggest the use of modern technologies with high sensitivity to low-velocity blood flow without the use of contrast agents, such as “microflow imaging” (MFI) on Philips devices, “superb microvascular imaging” (SMI) on Toshiba devices, “B-flow2 on General Electric devices. These devices allow for a better visualization of the residual lumen and contours of atherosclerotic plaques [17,18,19].

One error that must be avoided in PSV sampling is the “aliasing artifact” that is recorded for velocities that are too high; continuous-wave Doppler rather than pulsed Doppler should be used in these cases, because of its better temporal resolution on flow.

Particular attention should be paid in the case of patients with well-known contralateral severe stenosis, where the ipsilateral color Doppler study might be distorted by an overestimation of the degree of stenosis due to increased PSV values; in these cases, one solution might be to discern the compensatory increase in flow rate from the ratio of the peak velocities in the contralateral CCA and ICA.

Elevated carotid bifurcation, obesity, extensive vascular calcification, or in situ endovascular stents create difficulties in recording velocity readings by distorting the obtained data, resulting in an unreal increase in blood flow velocity. Elevated systolic BP, severe aortic valve insufficiency, and reduced cardiac output also affect the recorded blood flow velocities, leading to potential overestimation pitfalls, which is a limitation of US [20].

Therefore, US may not be able to accurately distinguish between partial and complete vascular occlusion, although the distinction is critical in a clinical setting [9].

### 2.2. CEUS

Contrast-enhanced ultrasound (CEUS) represents a diagnostic improvement performed as a completion of a conventional US exam and requires intravenous contrast agent use; to date, SONOVUE is the most used contrast agent for clinical diagnostic applications and consists of microbubbles of sulfur hexasulfide encapsulated inside a phospholipidic shell [7].

The microbubbles have an average diameter of 2.5 μm, which allows them to flow through the entire vascular bed, reaching the smallest capillaries and crossing the pulmonary circulation, but never crossing the endothelium and exiting the vascular lumen; so, they are strictly intravascular contrast agents.

SONOVUE is metabolized at the hepatic level (the phospholipidic component of the outer shell) and eliminated by the pulmonary/aerial route (internal sulfur hexafluoride); for this reason, it is safe in patients with renal failure and also causes a very low percentage of allergic reactions.

The absolute contraindications, however, are acute heart failure and allergy to the contrast agent [21,22].

CEUS applications in carotid stenosis studies provide information both at the macrovascular level, detecting plaque irregularities and possible ulcerative phenomena, and at the microvascular level, when studying intraplaque neovascularization [23].

The examination technique involves the use of a specific setting of US parameters, which can be corrected manually or preselected; particular attention deserves the mechanical index (MI), which is important to determine the power of the ultrasound beam. For conventional US techniques, the MI is generally higher than 1.6; for CEUS, however, a lower MI (0.03–0.04) should be used [24].

Currently, the most used CEUS modality is the “pulse-inversion technique” that allows us to selectively visualize the microbubbles’ echo, eliminating signals from static tissue. For this reason, CEUS is more accurate in assessing plaque surface and the luminal vascular edge, classifying them in smooth, irregular, or ulcerated, compared to echo-color Doppler and MDCTA [21].

In 18% of ulcerated plaques, a pathological sign detected by CEUS is the “microbubbles’ swirling movement”, which can also be observed by color Doppler US as the “yin–yang sign” [25].

CEUS can also allow for detecting an intraluminal thrombus thanks to a typical circumferential microbubble redistribution around the thrombus, which, in axial scans, appears as the “donut sign” [26].

Moreover, the “reperfusion technique” allows us to quantify the arrival, in the imaging field, of new microbubbles after emitting an instantly high MI pulse to interrupt them; in this away, it is possible to estimate intraplaque neovascularization [27].

By a histopathological correlation of intraplaque enhancement findings on CEUS, the neovascularization grade can be divided into four visual levels. Grade 0 is defined as the absence of intraplaque improvement; Grade 1 is defined as limited potentiation; Grade 2 as moderate potentiation; and Grade 3 as the presence of pulsating arterial vessels on CEUS imaging [28].

A long scan capacity of the contrast agent and a high spatio-temporal resolution provide a real-time scan pattern of a plaque and represent CEUS advantages with respect to CT and MRI; moreover, they offer the possibility to study both carotid arteries using a single SONOVUE dose [27,29].

The main limitations of this technique are artifacts generated by extensive parietal calcifications, a low panoramic view, and the operator’s limited experience [30].

### 2.3. New Ultrasound Developments: 3D US and Vector Flow

**Figure 1:**
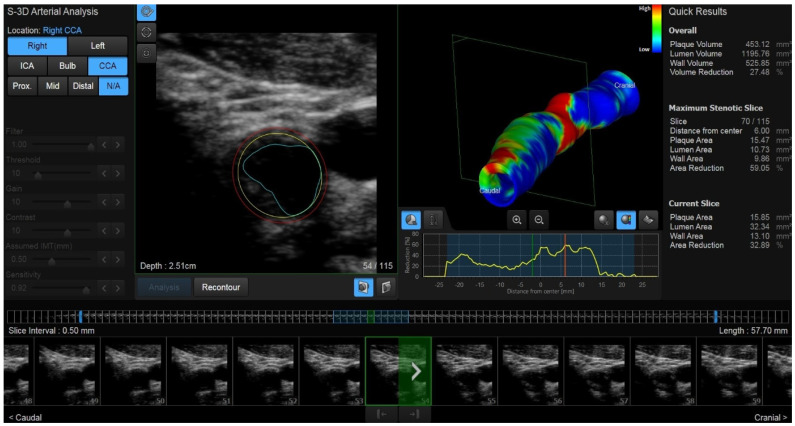
Shown is the 3D US reconstruction of a nonsignificant eccentric fibrolipid plaque.

Nowadays, high-flow US and 3D US (Figure 1) are the most advanced radiological techniques for morphological and functional parameter evaluation, resulting necessary in predicting plaque vulnerability; in particular wall shear stress (WSS) estimation appears more accurate with 3D US than with high-flow US [31].

An atherosclerotic plaque is an irregular three-dimensional phenomenon; so, 3D US has been shown to be more sensitive than 2D US for volume quantification during plaque evaluation, especially in the case of a clinically important stenosis in symptomatic patients. It is possible to approximatively calculate the plaque volume by obtaining consecutive serial axial scans of the affected vessel at 1–2 mm intervals and summing all sections obtained; in this way, plaque volume should represent the best morphological parameter to predict the future breakage risk [32,33].

Furthermore, it is possible to estimate even the total ulcer volume, which is predictive of the risk of acute cerebrovascular events [34].

Additionally, compared to conventional US, 3D US is superior in differentiating ulcerations from parietal discontinuities existing between consecutive plaques and in detecting changes in ulcer morphology.

Three-dimensional ultrasonic characterization of tissue can also provide more complete information when assessing atherosclerotic burden and plaque volume: unfortunately, inhomogeneity and other features such as hemorrhage and the presence of a lipid core cannot be assessed with US because they both appear hypoechogenic; so, they need to be studied with MRA to identify patients at higher risk of future events [35].

Vector flow (VF) assessment, on the other hand, is an emerging ultrasound investigation that considers changes in vascular hemodynamics over time due to a plaque [31]. VF is a technology implemented exclusively on Mindray devices; so, it cannot be applied with devices produced by other ultrasound companies and, therefore, cannot be widely used in clinical practice.

VF can quantify, at the plaque level, a new ultrasound parameter defined as “wall shear stress” (WSS), representing the friction force exerted by the blood flow on each point of the endothelial atheroma surface. Technically, VF consists in a multidirectional excitation of the examined vessels using several plane mechanical waves to obtain WSS values [36].

Considering that a plaque is strongly affected by hemodynamic changes due to shear stress, WSS could became a determining factor in assessing plaque evolution and stratifying patient risk, even for asymptomatic patients.

High WSS values for an atherosclerotic surface are strictly indicative of plaque vulnerability and so strongly predictive of future complications related to thromboembolic events [37].

Unlike B-mode and color Doppler US, VF is independent of the ultrasound angle; therefore, VF uses a high frame rate to track high speeds in real time and to intercept transient flow phenomena resulting in a high space–time resolution [36].

In combination with CEUS, VF improves image quality by increasing the vascular pool echogenicity provided by microbubbles.

## 3. CTA

**Figure 2:**
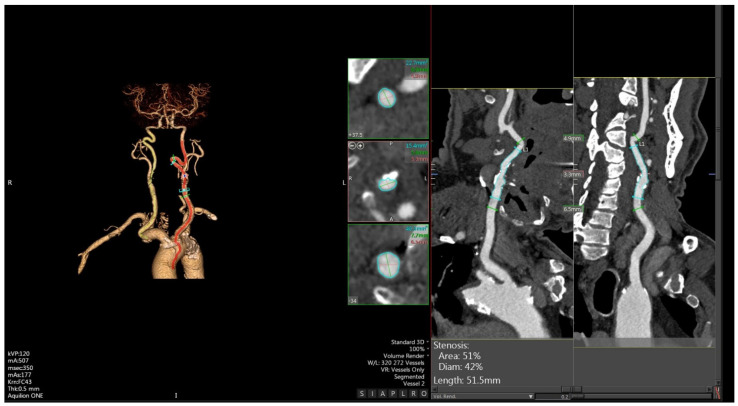
Shown are 2D and 3D reconstructions of a nonsignificant calcific plaque by CTA.

CTA (Figure 2) represents a second-level diagnostic imaging test to evaluate carotid plaques.

It is optimal to accurately assess the morphology and the stenosis grade of a vessel lumen, applying well-known measurement systems. It has excellent inter-operator reliability, high sensitivity (98%), and a positive predictive value of 93% [6,38].

Unlike US, CTA examinations are relative standardized between platforms and institutions and let the operator evaluate both carotid arteries simultaneously, either in the extracranial or in the intracranial tract, as well as the cerebral parenchymal flow. For these reasons, it is extremely useful both in acute stroke decision-making and in cerebrovascular prevention [3,6].

CTA has also been shown to have greater sensitivity and specificity in detecting ulcerations and plaque clots with respect to digital subtraction angiography (DSA) and US. It also detects and quantifies intraplaque neovascularization based on a proportional, increased contrast uptake.

However, CTA has limitations: it does not allow for hemodynamics studies and so has a low space–time resolution, involves the use of ionizing radiation, and requires a nephrotoxic contrast agent injection; in addition, in the presence of strongly calcified lesions, it can overestimate the plaque load [39].

Nevertheless, it is still often used because it is widely available, not susceptible to movement artifacts, especially in non-cooperative patients, and provides a wide overview and better anatomical details for therapeutic planning [6].

Furthermore, new scientific evidence focuses on the measurement by CTA of the total volume of a plaque. This parameter seems to be important because it is correlated with the increase in the vulnerability of the plaque and therefore with the incidence of the risk of stroke; in addition, it is useful in predicting cardiovascular outcomes in a more reliable way than both IMT and total plaque area [40,41].

The analysis of the total plaque volume is enabled by new reconstruction intelligence software applied to divide the plaque volume into multiple subcomponents, different in morphology and composition, and thus offers the possibility of precisely quantifying the volume only in the segment of interest [42].

The sub-component estimation allows for identifying even the intraplaque density, recognizing five different thresholds for the various components of a plaque: calcium (>130 HU), fibrous tissue (60 to 130 HU), lipid tissues (<60 HU), lipid IPH (26 to 59 HU), and IPH (<25 AU) [43]. Regarding the fibrous cap, a higher post-contrast enhancement is recorded in the case of cracking.

These new CTA studies can provide equal or even better results in detecting soft tissue sub-components of plaque than MRI. It is not only the overall plate volume that can predict plaque breakage, but also the relative percentages of the sub-components.

Finally, CT represents the unique alternative in those patients who have contraindications to MRI (e.g., incompatible prostheses or MRI pacemakers) [3].

## 4. MRI

**Figure 3:**
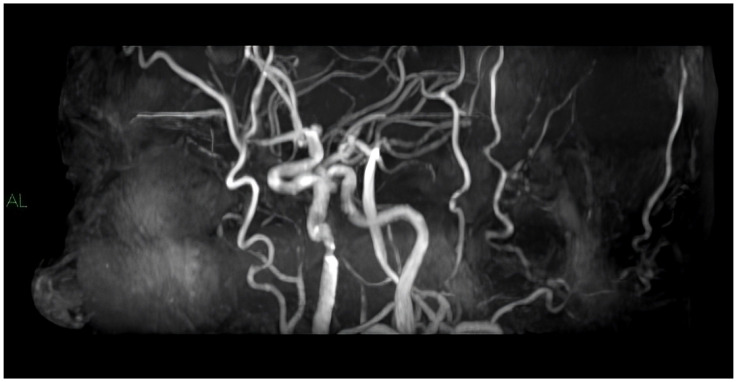
Shown is a 3D MRI reconstruction of a carotid stenosis by a time-of-flight sequence without the use of a contrast medium.

MRI (Figure 3) represents a second-level method for the study of carotid stenosis; in particular, current developments suggest the use of high-field superconducting magnets [6].

It has proven accurate in delineating the morphology of a plaque, its components, and its total load. This is also thanks to the introduction of surface coils with a limited field of view, which return high-resolution images, optimizing the signal/noise ratio at the carotid bifurcation compared to standard dedicated coils [44,45,46,47,48].

As regards the MRI technique, two types of sequences are used: “black blood” sequences, T1-, T2- and DP-weighted, and “white blood” sequences such as TOF (time of flight), which do not involve the use of a paramagnetic contrast and CE-MRA (contrast-enhanced magnetic resonance angiography), T1-weighted sequences [49,50,51].

“White blood” sequences are employed in order to study the vasal lumen, being able to evidence phenomena of ulceration and IPH; in particular, the TOF technique does not require the use of a paramagnetic contrast agent, which is safe in patients with renal failure or who reported a previous allergic reaction to the contrast agent, although CE-MRA is more accurate and, unlike the TOF technique, allows for also evaluating intraplaque neo-vascularization. On the other hand, these sequences do not allow for a good evaluation of the vasal wall because they are strongly affected by artifacts due to the attenuation caused by adjacent tissues.

Particularly interesting is the MPRAGE (Magnetization-Prepared Rapid Gradient Eco) sequence, heavily weighted in T1, which results the best to discern IPH thanks to the suppression of fibrous tissue and fat signals.

“Black blood” sequences, instead, are characterized by the subtraction of the intraluminal signal and appear, therefore, optimal for the evaluation of the vasal wall, being able to distinguish the different wall sub-components (IPH, LNRH, fibrous cap, calcifications) [44,52].

Therefore, overall, the association of these MRI sequences allows for studying with good reproducibility and reliability the characteristics involved in the determination of plaque vulnerability. For example, sensitivity and specificity are, respectively, 91% and 95% in detecting an LRNC and 94% and 97% in detecting IPH, while sensitivity in characterizing a fibrous cap is about 89% [53,54].

Moreover, since it does not use ionized radiation, differently from CTA, MRI appears more versatile and safer. We already pointed out that the wall shear stress (WSS) is involved in initiation, progression, and changes in plaque composition. In fact, the stress induced by blood pressure can exceed the resistance of the supporting tissues, being able to determine plaque breaking and the consequent thrombotic phenomena [55,56,57,58].

Thus, new developments in MRI have focused attention on new hemodynamic parameters such as the quantification of wall displacement and deformation over time in relation to the cardiac cycle. More detailed information on plaque composition can be derived from tissue strength data. In this regard, the WSS can be calculated using dynamic sequences such as those obtained with the Phase-Contrast Angiography (PCA) technique: in this way, one can evaluate the changes in direction of the WSS during the cardiac cycle by calculating the oscillatory cut index (OSI). The limitation is that the WSS values tend to be significantly underestimated when the shear stress values are too high [59,60,61,62,63].

About the displacement of the wall, PCA sequences with the dedicated “DENSE” protocol allow, thanks to post-processing processes that involve a reduction in the hemodynamic signal of the blood, for discerning the soft sub-components of a plaque, which suffer a great deformation of the wall, from the hardest ones (fibrotic or calcific) that, on the other hand, suffer a small deformation of the wall [64].

These detailed studies are possible thanks to the use of thin slab sequences such as 3D TOF, 3D MPRAGE, and 3D PCA sequences, which, compared to their 2D counterparts, provide a better Signal-to-Noise Ratio (SNR) and better image resolution and allow for multiple reconstructions thanks to the acquisition of an isotropic voxel. However, 3D techniques are more susceptible to motion artifacts due to the long acquisition times [44,65].

More recently, additional emerging hemodynamic parameters have been suggested, such as normalized localized flow helicity and wall transverse shear stress (trans-WSS); however, their applications are still very limited and need new developments in the future [66,67].

As regards the main limitations of MRI, they are certainly represented by the long acquisition times, the great susceptibility to moving artifacts (especially for sequences with low thicknesses), the not immediate availability, the high costs, the limited overview, and the low sensitivity and specificity in the detection of calcifications, which are 76% and 86%, respectively.

## 5. Discussion

Ischemic stroke is an acute cerebrovascular event that requires immediate intervention in emergency departments and the second leading cause of disability and mortality worldwide, with a prevalence of about 9.2%, increasing with age [68,69].

About 10 to 20% of ischemic strokes result as a complication of an underlying atheromatic carotid disease [1], which represents an inflammatory vascular pathology with a chronic–degenerative course. It is characterized by the formation of plaques in the arterial wall, which could cause lumen stenosis, and possible instability leading to plaque rupture and consequent athero-thrombotic and athero-embolic phenomena. It is estimated that about 816 million people between 30 and 79 years old have a carotid plaque, of which 58 million have a morphologically significant stenosis [70].

Radiology offers an optimal possibility for carotid plaque studies thanks to different investigation techniques. The main imaging methods used are US, CTA, and MRA, useful both in the morphological evaluation of plaques and in the research of possible vulnerability phenomena such as IPH, ulceration, neovascularization, fibrous cap, and LRNC, determining plaque instability and possible breakage [8].

The diagnostic sensitivity and specificity in detecting plaque instability of US, CTA, and MRA are 94%, 83%, and 100%, and 93%, 73%, and 89%, respectively [71].

Although modern developments have shown particular interest in the diagnostic possibilities offered by CTA and MRI, US remains the first-line method to assess plaque composition and stability and for the anticipation of future adverse events, since it has, compared to other imaging techniques, the ability to study plaque vulnerability also from a hemodynamic as well as from a morphological point of view.

In fact, new developments such as vector flow US focus on assessing the wall shear stress as a possible hemodynamic factor determining plaque instability: through the calculation of the WSS and the related wall deformation degree, we can obtain information about the composition of a plaque and its rupture risk and therefore determine the probability of future cerebrovascular events in subjects affected by carotid atheroma [37,59].

The true limit of US, in addition to those mentioned above such as the operator-dependence and the low panoramic view, appears in the presence of extensive calcium deposits at the plaque level that can affect the diagnostic reliability of US through various mechanisms, for example, through the formation of shadow cone artifacts that could block a complete visualization of the intimate–media interface by affecting the ability to detect plaque ulcerations or by causing compensatory hemodynamic changes in the blood flow at the level of the calcific plaque, which could provide falsified values for PVS and WSS [72].

CTA, thanks to the new high-performance machines that allow one to obtain very thin layer images with excellent spatial resolution and contrast and to perform 3D reformat, potentially represents the best method for the evaluation of carotid plaques, given the high panoramic view it provides, the objectivity of the extrapolated data, and the standardization of imaging parameters related to plaque morphology and vulnerability.

Nevertheless, CTA is not the most widely used imaging method for the study of plaque vulnerability in election regime because it requires the use of ionizing radiation and nephrotoxic contrast agents; but, on the other hand, it is the first-line technique for the assessment of acute cerebrovascular events resulting from an underlying plaque complication.

However, new TC developments have provided intelligent software that can recognize the various plaque sub-components and calculate their volume; in this way, it is possible to achieve a more accurate estimation of plaque vulnerability and instability [42].

Nowadays, MRA, despite not using ionizing radiation, is the least used imaging method among those mentioned, due to the long acquisition times that are often responsible for the appearance of movement artifacts, the limited capillary availability, the high costs, and the need to use high-field magnets to obtain images with excellent contrast resolution and therefore diagnostic ability.

Encouraging developments, however, have made it possible to re-evaluate MRA thanks to the possibility of studying plaque vulnerability through the flow hemodynamics, similarly to US. Specific sequences such as Phase-Contrast Angiography (PCA) sequences and dedicated protocols such as “DENSE” allow, in fact, not only for quantifying the WSS but also for evaluating the value fluctuations over time due to changes in the direction of the blood flow at the level of the plaque.

Therefore, the role of the radiologist becomes fundamental in the multidisciplinary management of patients with carotid plaque because, compared to the past, the radiologist can select those patients eligible for endovascular or surgical treatment, regardless of a significant stenosis degree, including, therefore, patients with asymptomatic plaques [8].

In this regard, US is the most widely used imaging method due to its wide availability, by low costs, good diagnostic accuracy, and the possibility of providing a wide spatio-temporal resolution. However, it remains a highly operator-dependent technique with low panoramicity.
